# Dynamic Testing of Materials for Galvanising Pot Roll Bearings with Improved Performance

**DOI:** 10.3390/ma17235837

**Published:** 2024-11-28

**Authors:** Giovanni Paolo Alparone, James Sullivan, Christopher Mills, James Edy, David Penney

**Affiliations:** 1 Department of Materials Engineering, Swansea University, Swansea SA2 8PP, UK; 2 Tata Steel, Research and Development, Swansea Technology Centre, Swansea SA2 8PP, UK

**Keywords:** ceramics, corrosion, wear testing, galvanising pot journal bearings, continuous galvanising, galvanising pot hardware

## Abstract

Galvanising pot roll bearings are subjected to severe deterioration due to the corrosion of the bearing materials in liquid Zn, resulting in maintenance stops that can cost thousands of pounds per hour in downtime. Dynamic wear testing in molten Zn-Al and Zn-Al-Mg was conducted to assess the corrosion and wear resistance of three material pairs using a bespoke testing rig. The materials investigated in this study were Wallex6^TM^ coated with WC-Co, stainless steel 316L coated with Al_2_O_3_, and as-received Wallex6^TM^ and Wallex4^TM^ alloys. It was found that only the Al_2_O_3_ coating remained unreactive in Zn alloy, whereas the materials containing Co were corroded, as evidenced by the formation of intermetallic compounds containing Al-Co-Zn-Fe. The results also highlighted that the dissolution of the Co matrix and diffusion of Zn and Al from the bath occurred in Wallex6^TM^ and Wallex4^TM^. However, the diffusion of Zn into the Wallex^TM^ alloys was reduced by approximately 60% in the Zn-Al-Mg bath compared to Zn-Al. The wear scars were analysed to determine the wear coefficient of the worn specimens. Out of the three material couplings investigated in this study, minimal wear damage in both Zn-Al and Zn-Al-Mg was only obtained by pairing Wallex6^TM^ with Al_2_O_3_ coatings.

## 1. Introduction

Continuous galvanising is a hot-dip process in which the strip steel is immersed in a bath containing liquid Zn alloy of the desired composition. Automotive customers require a ‘full finish’, high surface quality, which is important for visible parts that must be free of imperfections [1]. As a result, the galvanised steel sheet demanded by the automotive industry must have an excellent surface finish free from defects and with a controlled surface roughness [2]. Recently, the use of Zn-Al-Mg coatings has become popular in the automotive industry due to their potential to reduce fuel consumption via lightweighting, as they require a lower thickness compared to Zn-Al coatings [3]. In addition to this, automotive industries use Zn-Al-Mg coatings due to their excellent galling resistance [4,5].

During the immersion process in the hot-dip galvanising bath, the strip steel is guided by the pot hardware, which includes the pot rolls and the roll journal bearings. In order to produce galvanised products that meet the requirements of automotive customers, the stability of the strip steel through the pot of liquid Zn alloy is important. Vibrations in the strip, especially after exiting from the Zn pot and around the gas knives section of the line, must be minimised to produce a high-quality product, as the gas knives control the thickness of the coating. However, the materials of the pot roll bearing components, namely the journal sleeve and bushing, react with the molten Zn alloy at temperatures greater than 400 °C and the deterioration of the bearings induces vibrations in the strip as it passes through the gas knives section of the line [6,7,8,9]. Therefore, to avoid quality issues, the pot hardware must be changed and reconditioned, leading to downtime and loss of yield [10,11]. For this reason, increasing the lifetime of the bearings by suppressing the reactions of the bearing materials with the Zn bath will enable the production window of automotive-grade galvanised products to be extended, resulting in cost benefits for the galvanising industry.

The properties of ceramic materials have been studied for developing bearings with extended durability, due to their potential to resist the attacks of many molten metals at high temperatures [12,13,14,15,16]. In previous work, the corrosion behaviour of Al_2_O_3_ coatings was examined by conducting static immersion tests in liquid Zn-Al and Zn-Al-Mg [17]. The findings obtained in this study suggested that Al_2_O_3_ showed superior performance in Zn-Al, compared to materials that have been traditionally used to make hardware components, such as steel and Co-based alloys or coatings, which were found to be severely corroded by the molten metal [7,18,19].

However, the corrosion behaviour of these materials was assessed under static conditions. The behaviour of materials under dynamic conditions could differ from that observed during static testing. In the dynamic situation, the pot hardware was exposed to chemical attacks from the liquid metal and dynamic wear due to the sliding of the bearing materials under load. It was demonstrated that the corrosion rates of materials in molten metal can differ from those observed in static baths [20]. Previous studies attempted to carry out dynamic tests on materials with potential use as pot-bearing materials in continuous galvanising [21,22]. However, a limited selection of materials has been tested under dynamic conditions; in addition to this, there is no evidence of dynamic tests conducted in Zn-Al-Mg baths in the literature.

For this reason, the present work investigates the performance of three material couplings tested under dynamic conditions in Zn-Al and Zn-Al-Mg using a bespoke dynamic testing rig. These experiments were performed on a current configuration, which coupled WC-Co coatings with Wallex6^TM^ and two potential upgrades to the benchmark configuration. The first of these potential upgrades involved replacing Wallex6^TM^ with Wallex4^TM^ and the second potential upgrade consisted of pairing stainless steel (SS) 316L coated with Al_2_O_3_ and Wallex6^TM^.

## 2. Materials and Methods

### 2.1. Materials

The specimens used in this study consisted of 20 mm diameter × 150 mm-coated cylinders, which were tested against 25 × 50 × 10 mm pads. The three material couplings chosen for dynamic testing are summarised in Table 1. Wallex6^TM^ and Wallex4^TM^ were procured from Wall Colmonoy (Swansea, UK). WC-Co coatings were applied with a thickness of 150 µm. Al_2_O_3_ coatings were applied to SS 316L bars with a thickness of 250 µm via a high-velocity oxygen fuel (HVOF) thermal spray process, carried out externally by Engineered Performance Coatings (Cardiff, UK). The reason for choosing thermal spraying over other coating fabrication methods is that previous studies have shown that Al_2_O_3_ could be successfully deposited onto stainless steel [12]. The compositions of Wallex6^TM^ and Wallex4^TM^ were provided by the supplier and are found in Table 2.

### 2.2. Experimental Procedure

**Figure 1:**
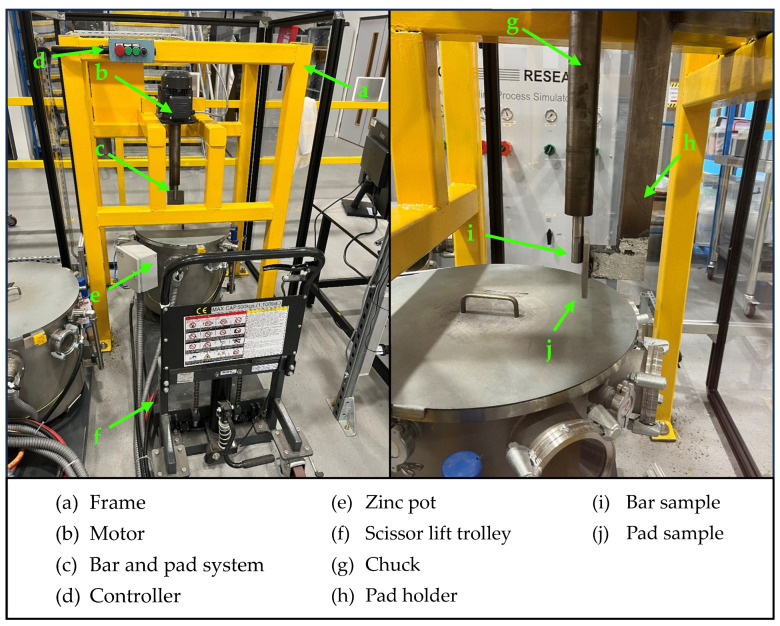
Bespoke dynamic testing rig showing the main components.

The material pairs listed in Table 1 were mounted on a bespoke dynamic testing rig, as shown in Figure 1. The rig enabled the testing of a round bar specimen that was mounted on a shaft connected to an electric motor. The bar was capable of rotating up to a speed of 300 RPM and it was allowed to slide against a static pad specimen with a force of 60 N. The rig was equipped with a heated Zn pot so that the two specimens could be submerged in molten metal. The pot was loaded with approximately 40 kg of Zn alloy ingots. Each material coupling was tested in Zn-0.3wt%Al, also known as GI, and Zn-1.5wt%Al-1.5wt%Mg bath compositions. The pot was set at a temperature of 465 °C, which was monitored using thermocouples installed inside the pot. As the Zn melted, dross formed on top of the bath, which was removed before starting the test. Once the Zn was fully molten, the pot was raised so that the bar was just above the Zn level and was held for 24 h to preheat the specimens and to minimise the effects of thermal shock. Subsequently, the pot was raised to submerge the bar and the pad in liquid Zn and the lid was closed. The bar was rotated in a clockwise direction at a speed of 300 RPM for 48 h. After the test, the pot was lowered, and the specimens were removed from the holders. Samples from the bar and the pad were cross-sectioned for characterisation. Images were captured using a ZEISS (Oberkochen, Germany) EVO scanning electron microscope (SEM) and a Hitachi (Tokyo, Japan) TM4000 desktop SEM, both equipped with backscattered electron detectors (BSD). Energy Dispersive X-ray analysis (EDS) was performed using an Oxford Instruments (Abingdon, UK) EDS detector with Aztec 6.1 software. The solidified Zn was removed with 35% HCl on the pad specimens, which required measurement of the volume loss. The displacement of the bar after 48 h of sliding was determined by measuring the depth of the wear scar left on the pad specimen. The 3D maps of the wear scar were generated using the Keyence VHX-7000 (Osaka, Japan) digital microscope. The volume loss ‘*V*’ was obtained from the cross-sectional area and the length of the scar according to Equation (1):

(1) V=A×L,

where ‘*A*’ is the measured cross-sectional area of the wear scar and ‘*L*’ is the length of the specimen. The worn track section ‘*A*’ was determined using the digital microscope. The total sliding distance ‘*S*’ was obtained by multiplying the sliding speed ‘*v*’ in [m/min] and the duration of sliding ‘*t*’ in [min] (Equation (2)):

(2) S=v×t,

This value was subsequently used to calculate the wear coefficient ‘*k*’, as shown by Equation (3) [23]:

(3) $$k=\frac{V}{F\times S\;}[{\mathrm{mm}}^{3}/\mathrm{Nm}],$$

where ‘*F*’ is the contact load and ‘*S*’ is the sliding distance.

## 3. Results and Discussion

### 3.1. Imaging of As-Received Samples

**Figure 2:**
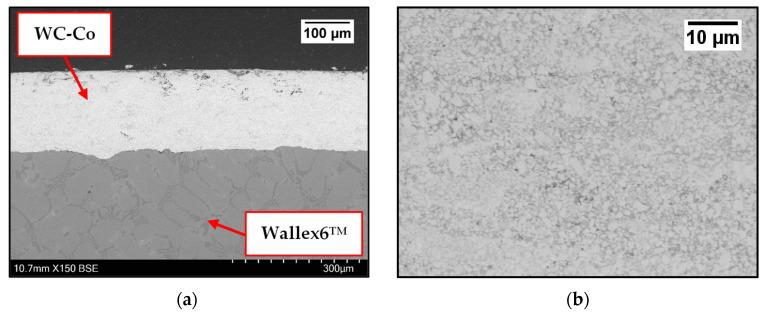
Cross-section of the as-received Wallex6^TM^ bar coated with WC-Co (**a**); details of the WC-Co coating (**b**). Please note the different magnifications.

**Figure 3:**
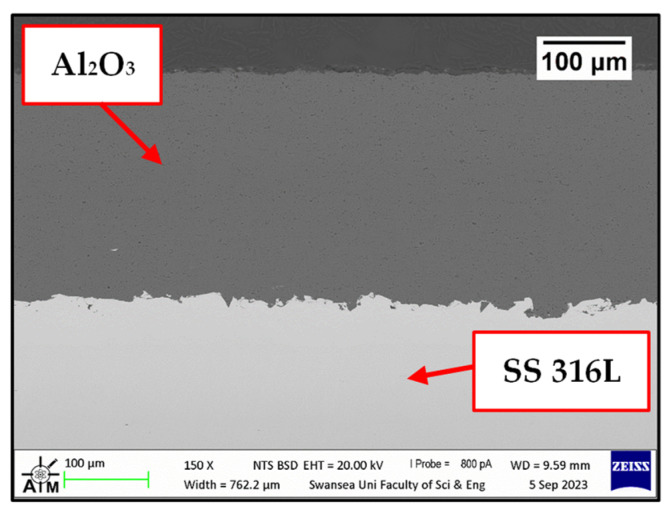
SEM image of the cross-section of high-velocity oxygen fuel (HVOF) Al_2_O_3_ coated onto the surface of stainless steel (SS) 316 L.

**Figure 4:**
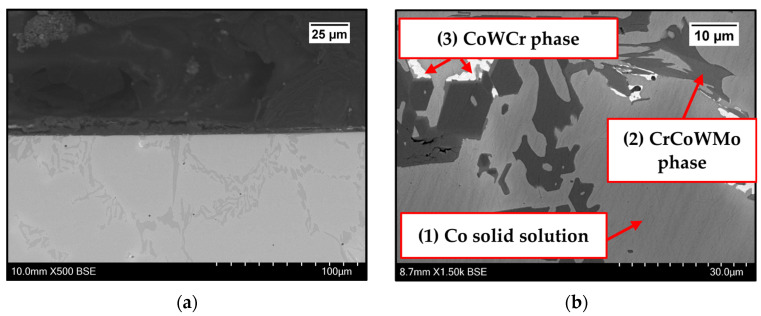
SEM images of the cross-sections of Wallex6^TM^ captured at the interface (**a**) and in the bulk of the material (**b**). Please note the different magnifications.

**Figure 5:**
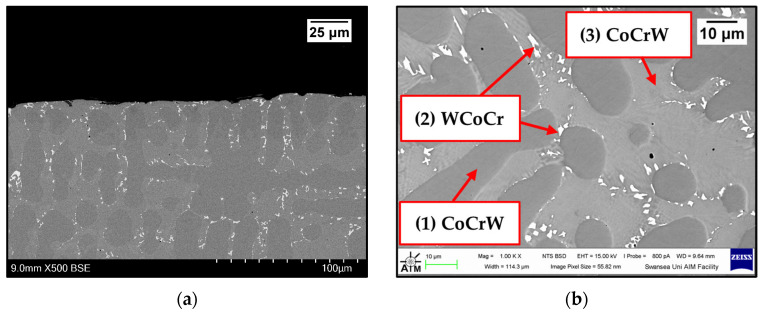
SEM images of the cross-sections of Wallex4^TM^ captured at the interface (**a**) and in the bulk of the material (**b**). Please note the different magnifications.

Images were taken on the as-received samples before exposure to Zn-Al and Zn-Al-Mg. Cross-sections were captured on as-received WC-Co/Wallex6^TM^ (Figure 2a,b) and Al_2_O_3_/SS 316L bar specimens (Figure 3). Figure 2b shows the microstructure of WC-Co coatings, which is characterised by WC grains (bright contrast) surrounded by the Co binder phase (dark contrast) [24]. The microstructure of Al_2_O_3_ coatings was described in a previous work, where the same coatings were tested under static conditions [17]. The as-received pad specimens, namely Wallex6^TM^ and Wallex4^TM^, are shown in Figure 4a,b and Figure 5a,b respectively.

**Figure 6:**
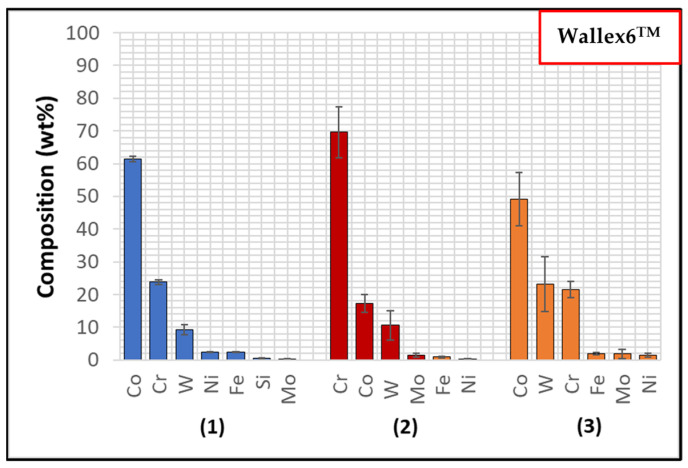
EDS phase elemental analysis of Wallex6^TM^. The numbers refer to the phases in Figure 4, where (1) is the Co solid solution phase, (2) is the CrCoWMo phase and (3) the CoWCr phase.

Wallex6^TM^ is the equivalent of Stellite 6 and is characterised by a Co solid solution matrix phase (1), with Co, Cr, and W as the main constituents. EDS found that the average element composition of this phase was Co (61.3 ± 0.8 wt.%), Cr (23.8 ± 0.6 wt.%), and W (9.3 ± 1.5 wt.%), as shown in Figure 6. Two eutectic phases were observed in the Co matrix phase: a primary CrCoWMo (2) phase and a secondary eutectic phase of CoWCr (3) (Figure 4b). The results of EDS elemental analysis showed that the primary eutectic phase was composed of Cr (69.6 ± 7.8 wt.%), Co (17.3 ± 2.8 wt.%), W (10.6 ± 4.6 wt.%), and Mo (1.4 ± Mo wt.%), with traces of Fe and Ni. The secondary phase mainly contained Co (49.2 ± 8.1 wt.%), W (23.2 ± 8.4 wt.%) and Cr (21.6 ± 2.5 wt.%).

**Figure 7:**
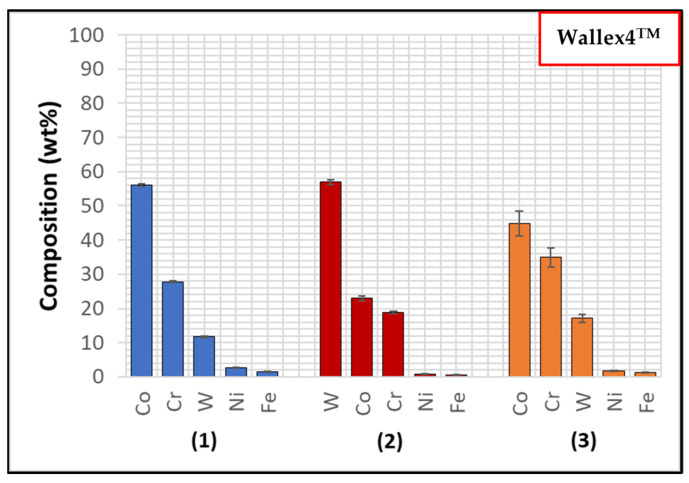
EDS phase elemental analysis of Wallex4^TM^. The numbers refer to the phases in Figure 5, where (1) is the CoCrW phase, (2) is the WCoCr phase and (3) the CoCrW phase.

The composition of Wallex4^TM^ is similar to that of Wallex6^TM^. The SEM image showed the presence of three different phases (Figure 5b). EDS analysis was conducted to determine the composition of these phases, as shown in Figure 7. EDS revealed the presence of a dark grey phase (1), that is a solid solution matrix phase containing Co (56.1 ± 0.4 wt.%), Cr (27.8 ± 0.2 wt.%), W (11.8 ± 0.2 wt.%), Ni (2.7 ± 0.2 wt.%), and Fe (1.6 ± 0.1 wt.%). In addition to this, two eutectic phases were present. The bright phase (2) was made of W (57.0 ± 0.7 wt.%), Co (23.0 ± 0.7 wt.%), Cr (18.8 ± 0.2 wt.%), Ni (0.8 ± 0.0 wt.%), and Fe (1.3 ± 0.1 wt.%), whereas the light grey phase (3) contained Co (44.9 ± 3.6 wt.%), Cr (34.9 ± 2.7 wt.%), W (17.1 ± 1.1 wt.%), Ni (1.8 ± 0.1 wt.%), and Fe (1.3 ± 0.1 wt.%). It was observed that the bright eutectic phase had a greater portion of W compared to the Co-rich matrix; in addition to this, the composition of the dark grey phase was similar to that of the matrix, with greater Cr and W content. The composition does not significantly differ from Wallex6^TM^, as shown in Table 2; however, Wallex4^TM^ was found to have lower Co content (47.2 wt.%) and higher W additions (14.3 wt.%) compared to Wallex6^TM^, which contained Co (59.1 wt.%) and W (3.8 wt.%).

### 3.2. Dynamic Corrosion Testing

#### 3.2.1. Wallex6^TM^ with HVOF WC-Co and Wallex6^TM^

**Figure 8:**
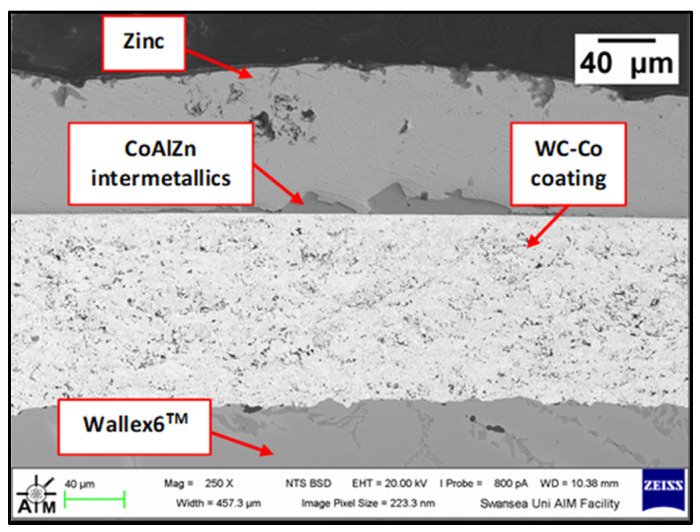
SEM image of the WC-Co/Wallex6^TM^ bar after testing in Zn-Al.

Figure 8 shows a cross-section of the WC-Co/Wallex6^TM^ bar after 48 h of testing in Zn-Al. The SEM image showed that the coating was not prone to damage after contact with the static pad specimen. Cracks, as well as pores, can provide pathways for liquid Zn alloy to penetrate the coating and corrode the base metal [16]. However, no reaction products accumulated below the coating, highlighting that Zn did not diffuse into the base metal. The integrity of the coating upon sliding with the Wallex6^TM^ counterpart was linked with the hardness of the material. WC is harder than Wallex6^TM^; it is known that the hardness of spherical WC lies between 2100–2500 HV, which is significantly higher than the hardness of Wallex6^TM^ (400 HV) and Wallex4^TM^ (570 HV) [25], suggesting that the Wallex^TM^ pads could not inflict severe wear damage to the bar specimens.

**Figure 9:**
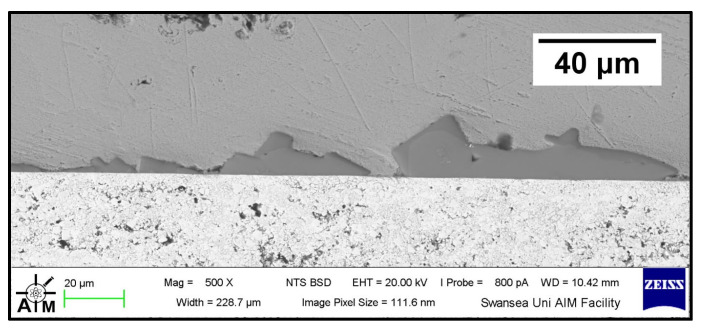
Higher magnification image of the intermetallic phases observed on the WC-Co coating after testing in Zn-Al.

**Figure 10:**
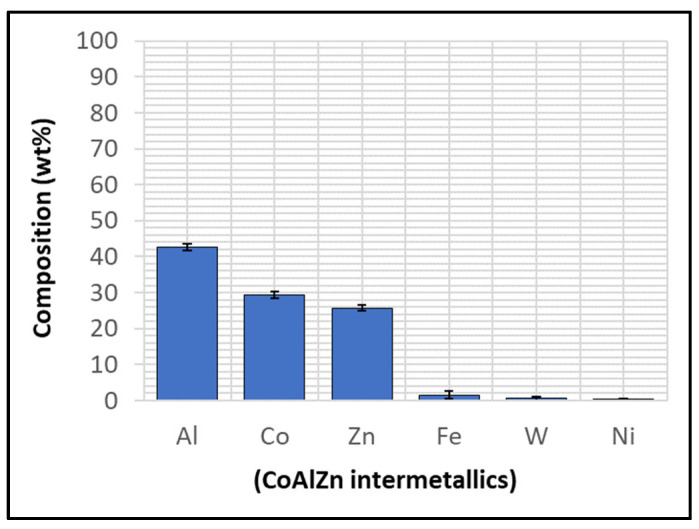
EDS phase elemental analysis of the intermetallic compounds on the WC-Co coating shown in Figure 9.

Although the WC-Co coating protected Wallex6^TM^ from the attack of liquid Zn-Al, the analysis of the cross-section revealed that intermetallic compounds formed on the contact surface, as illustrated in the high magnification image (Figure 9). These compounds were absent in the as-received specimen (Figure 2a). The results of the EDS phase elemental analysis conducted in this region (Figure 10) showed that the intermetallic compounds are Zn-based phases, which also contain Al (42.6 ± 0.9 wt.%) and Co (29.3 ± 0.9 wt.%), with traces of Fe, W, and Ni. Previous studies reported the presence of intermetallic phases on the surface of WC-Co coatings [26,27].

Co is known to have a strong affinity for Al present in the melt and, for this reason, Co-Al particles can develop in liquid Zn baths containing Al [28]. In the present study, Co is contained in the WC-Co coating of the bar specimen, as well as in the Wallex6^TM^ pad. Co-rich surfaces are known for being ideal sites for the attachment of intermetallic compounds existing in the liquid metal bath [28,29]. Experiments were performed by Zhang [29] on Stellite 6 bearings in GI, and it was found that Co-based aluminides were present on the bushing surface as a result of the reaction between the wear particles generated during testing and the Zn alloy bath. Therefore, the presence of aluminides illustrated in Figure 9 could be related to the adhesion of these particles to the coating surface. However, the formation of Al-Co-Zn intermetallic compounds in WC-Co coatings is often the result of the corrosion of the Co-rich matrix phase in the coatings by the Zn bath. As discussed by Seong et al. [26] and Tani et al. [27], Co reacted with the Al present in the Zn bath due to Co dissolution into the melt, leading to the formation of Al-rich compounds containing Co and Zn.

**Figure 11:**
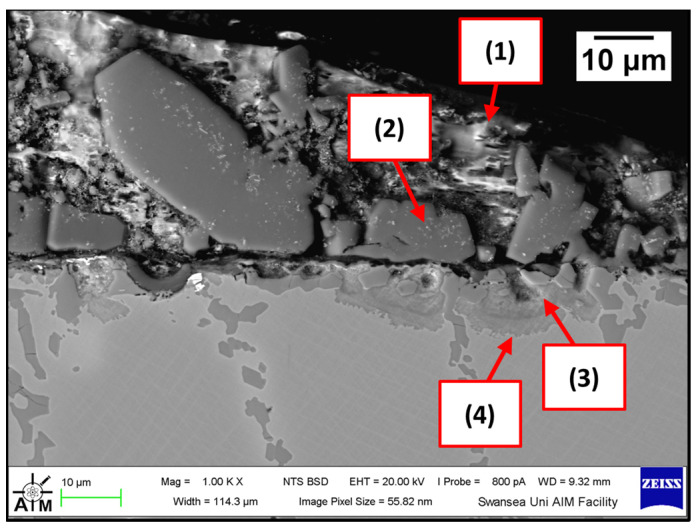
Cross-section of Wallex6^TM^ pad after testing in Zn-Al: (1) Zn phase, (2) intermetallic particles, (3,4) diffusion layer.

**Figure 12:**
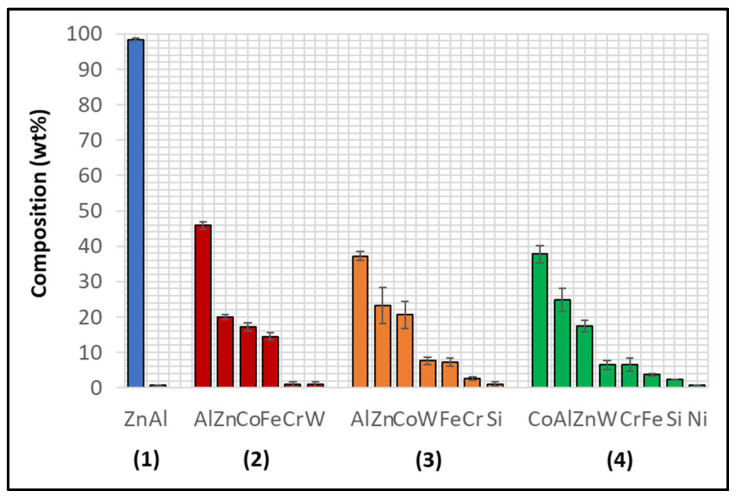
EDS point spectrum analysis of the phases present in the Wallex6^TM^ pad after testing in Zn-Al. The numbers refer to the phases shown in Figure 11, namely the (1) Zn phase, (2) intermetallic particles and (3,4) diffusion layer.

The Wallex6^TM^ pad was found to react with the molten metal bath, after exposure to Zn-Al. Cross-sections of the specimens were analysed in the unworn region (Figure 11) and they were compared to the as-received specimen (Figure 4). EDS point spectrum analysis was performed to reveal the composition of the phases observed in this area (Figure 12). The results clearly showed the presence of dross phases (2) within the top Zn phase (1) and on top of the surface in contact with molten Zn-Al. These Zn-based intermetallic compounds were found to mainly contain Al (45.9 ± 1.0 wt.%), Co (17.1 ± 1.2 wt.%), Fe (14.5 ± 1.0 wt.%) and small amounts of Cr and W. Co has a strong affinity for Al and there is evidence that the Co solid solution phase of Co-Cr-W alloys reacts with the Al contained in liquid Zn baths [30]. The products of this reaction are CoAl particles forming on the sample surface. The information obtained in the present study showed that Fe was contained in the CoAl intermetallic compounds after testing in Zn-Al. This finding aligns with previous studies, which reported that CoAl particles transformed to FeAl complexes, as the exposure to liquid Zn alloy increased because of the presence of dissolved Fe in the Zn alloy [26,28].

A reaction layer developed beneath the surface of the sample that was found to mainly contain Al-Zn-Co-Fe-Cr-W. Similar findings were obtained by Zhang [29] who reported the formation of a layer of cobalt-based aluminides of Al-Co-Zn-Cr-Fe-W on the contact surface of a Stellite 6 bearing after exposure to GI. In this study, the reaction layer was interrupted by the CoCrWMo eutectic phases present in the alloy, meaning that the diffusion layer did not form at locations where the eutectic phases were present. In Figure 11, (3) and (4), respectively, denote the region within the subsurface reaction layer and the interface between the reaction layer and the bulk of the alloy. It was observed that Al was present in the reaction layer, whereas the Co content diminished (20.6 ± 3.8 wt.%) compared to the unreacted Co-solid solution phase, as shown in Figure 6 (56.1 ± 0.4 wt.%). The Co content was found to increase (37.7 ± 2.4 wt.%) in the vicinity of the interface with the Co-solid solution phase, indicating that Co was depleted from the reaction layer below the surface.

**Figure 13:**
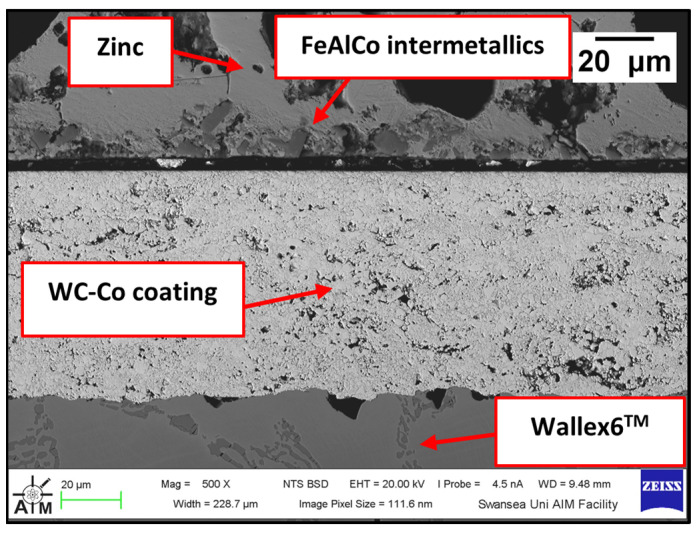
Cross-section of the WC-Co/Wallex6^TM^ bar specimen after exposure to Zn-Al-Mg.

**Figure 14:**
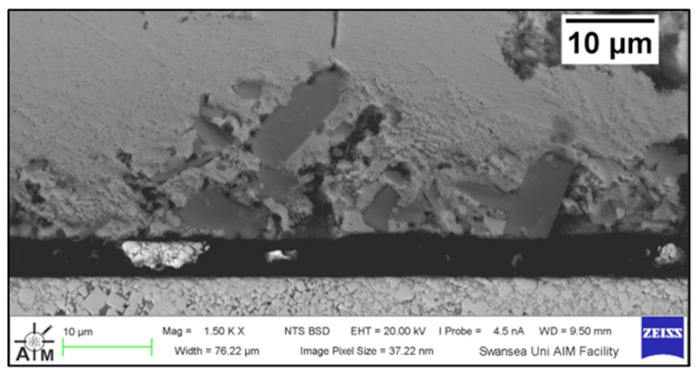
Higher magnification image of the intermetallic phases observed on the WC-Co coating after testing in Zn-Al-Mg.

**Figure 15:**
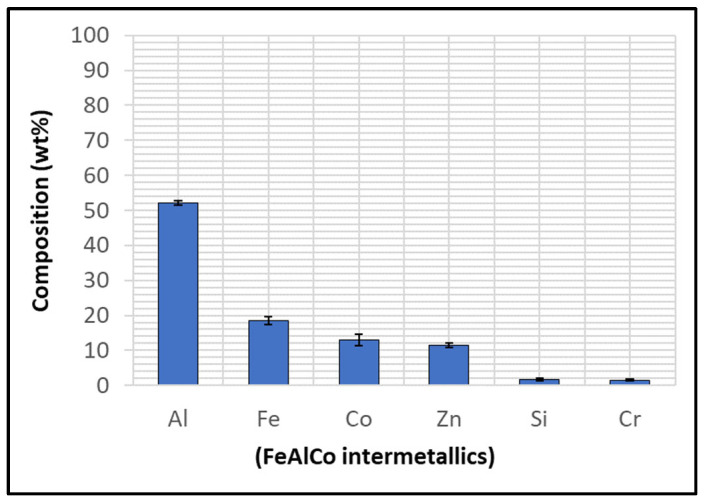
EDS phase elemental analysis of the intermetallic compounds observed on the WC-Co coating after exposure to Zn-Al-Mg.

The experiment was repeated on as-received specimens in Zn-Al-Mg. The cross-section of the WC-Co/Wallex6^TM^ bar specimen was imaged (Figure 13) and it was observed that particles of intermetallic compounds were present (Figure 14), showing similar behaviour to Zn-Al. However, the results of EDS analysis (Figure 15) revealed that these particles contained more Fe (18.5 ± 1.2 wt.%) than Co (12.9 ± 1.6 wt.%), whereas the intermetallic particles grown in Zn-Al were richer in Co.

**Figure 16:**
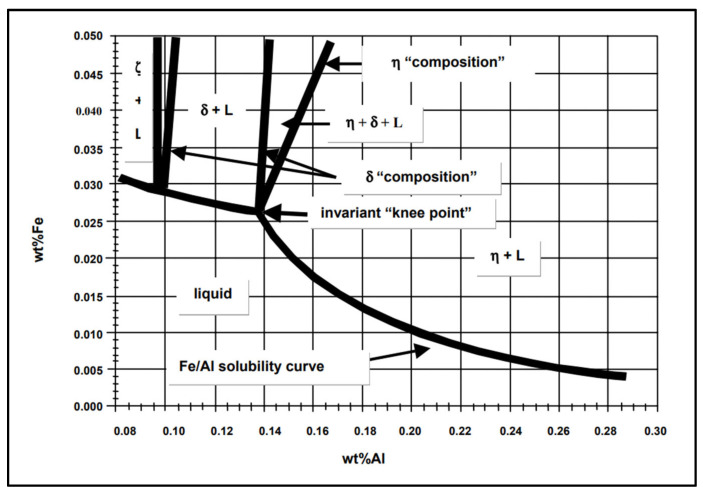
Phase diagram showing the Zn-rich corner of the Zn-Al-Fe system at 460 °C. The diagram illustrates the Fe solubility limit for Al concentrations up to ~0.3 wt.% Al [35].

The present literature highlights that the solubility of Fe in liquid Zn alloy depends on the Al content. In this experiment, the solubility limit corresponds to the maximum quantity of Fe that can dissolve in the Zn bath with the addition of Al or Al and Mg. The solubility limit of Fe in Zn-Al was determined from the experiments conducted by Tang [31] and it is a function of temperature [32]. Figure 16 shows a phase diagram constructed for the Zn-rich corner of the Zn-Al-Fe system, based on the more recent experiments conducted by McDermid*,* et al. [33].

The diagram shows the Fe solubility limits for Al concentrations up to approximately 0.3 wt.% at 460 °C. The area under the Fe/Al solubility curve indicates the region where Fe and Al remain in solution. It can be observed that this area decreases as the Al content in the bath is increased. Intermetallic dross compounds between Fe and Al do not form below the solubility limit. However, FeAl intermetallic phases form when the Fe solubility is exceeded [9,34]. Al tends to react with Co dissolved in the melt at low Al contents [18]. Therefore, the strong affinity of Co for Al could explain the higher Co content in the intermetallic phases observed for the Zn-Al bath with 0.3 wt.% Al.

The Zn-Al-Mg bath contains 1.5 wt.% Al and 1.5 wt.% Mg and it is reported that the presence of Mg does not significantly alter the Fe/Al solubility in the phase diagram. The area under the solubility line remains small as the Al content is increased from the Zn-0.3wt.%Al composition to the levels encountered in the Zn-Al-Mg bath and the Fe solubility is even smaller at higher Al content [34]. This information is supported by the results reported in the present study, which showed that the Fe content in the intermetallic phases detected after immersion in Zn-Al-Mg was higher by 17.1 wt.%.

**Figure 17:**
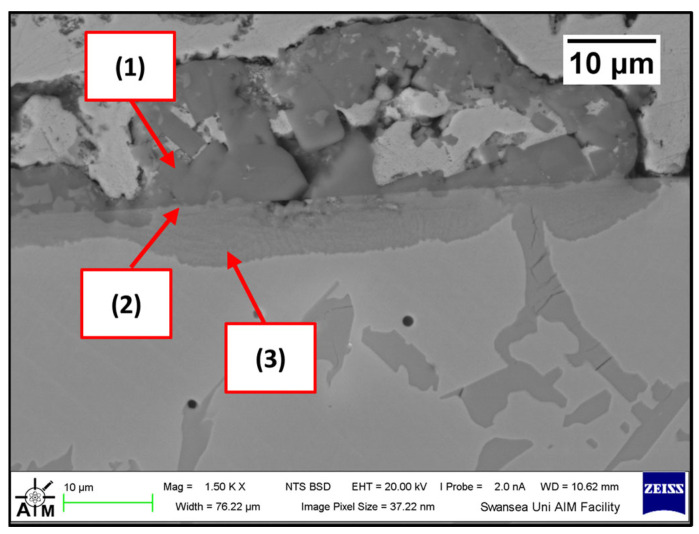
Cross-section of the Wallex6^TM^ pad specimen after exposure to Zn-Al-Mg: (1) intermetallic particles, (2,3) diffusion layer.

**Figure 18:**
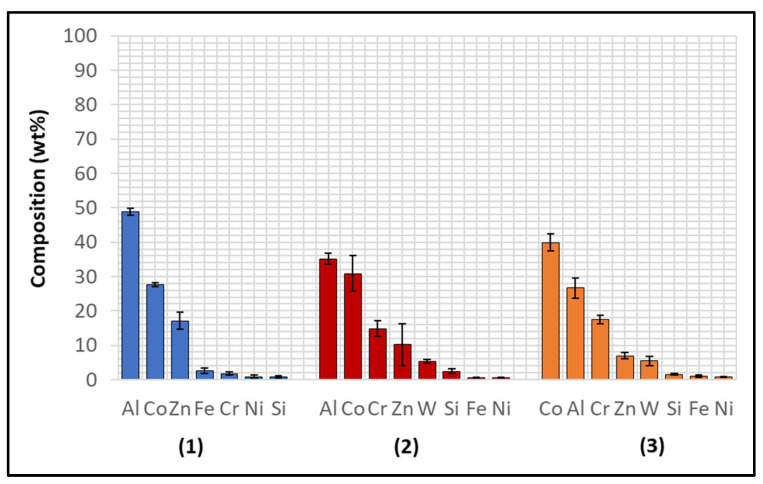
EDS point spectrum analysis of the phases present in Wallex6^TM^ after exposure to Zn-Al-Mg. The numbers refer to the phases shown in Figure 17, namely the (1) intermetallic particles and (2,3) diffusion layer.

The Wallex6^TM^ pad was corroded in a similar fashion to the specimen immersed in Zn-Al (Figure 17). CoAlZn intermetallic compounds grew on the surface of the material (1) and a diffusion layer formed beneath the surface of the sample (2). The results of the EDS analysis (Figure 18) showed that this reaction layer contained Al (35.1 ± 1.5 wt.%) and Co (30.9 ± 5.3 wt.%), whereas, outside this layer, the composition changed to Co (39.9 ± 2.4 wt.%) and Al (26.6 ± 2.9 wt.%), indicating Co depletion from the reaction layer, as observed after exposure to the Zn-Al bath, confirming that the introduction of Mg into the bath did not alter the corrosion behaviour of the materials present in the current configuration.

#### 3.2.2. Wallex6^TM^ with HVOF WC-Co and Wallex4^TM^

**Figure 19:**
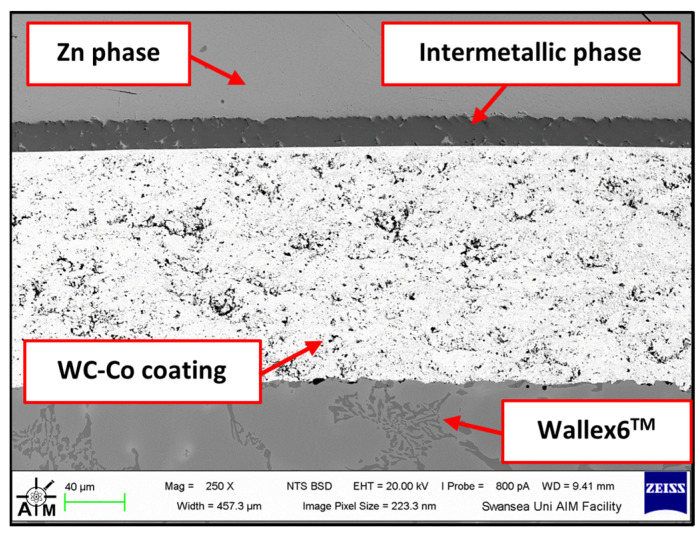
Cross-section of the WC-Co/Wallex6^TM^ bar after dynamic testing against Wallex4^TM^ in Zn-Al.

**Figure 20:**
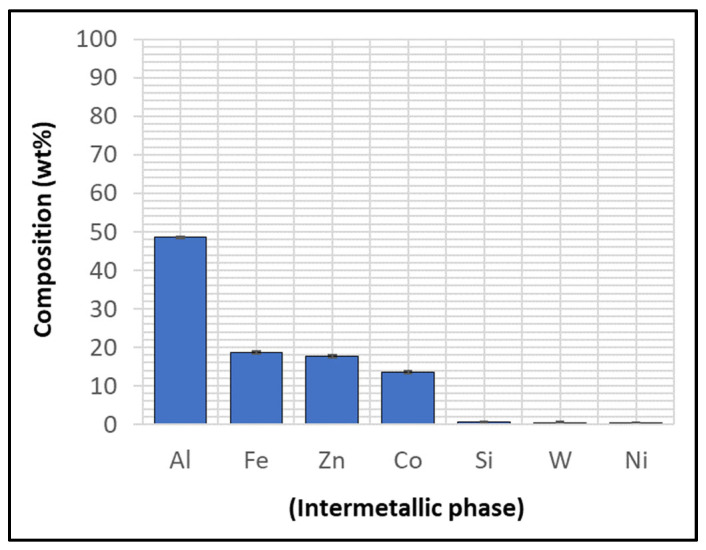
Composition of the intermetallic phase present in the WC-Co coating after dynamic testing with Wallex4^TM^ in Zn-Al.

The first of the two potential upgrades to the benchmark configuration involved replacing Wallex6^TM^ with Wallex4^TM^. The bar specimen used in the experiment was again coated with WC-Co applied via HVOF, as described in Section 2, *Materials and Methods*. Figure 19 was captured on the WC-Co/Wallex6^TM^ bar after sliding against the Wallex4^TM^ pad in Zn-Al. Similar behaviour to the bar specimens analysed previously can be observed. The Wallex6^TM^ base metal remained unreactive in Zn-Al, as the WC-Co coating prevented exposure to the molten metal bath. No cracks or other signs of damage to the coating were observed after the experiment. In a similar fashion to the coatings discussed in the previous section, the image was compared to the as-received specimen (Figure 2) and it was observed that a layer of intermetallic particles formed on the surface. The EDS phase elemental analysis (Figure 20) revealed the composition of the intermetallic particles, which contained Al (48.6 ± 0.2 wt.%), Fe (18.6 ± 0.4 wt.%), Zn (17.6 ± 0.3 wt.%), and Co (13.5 ± 0.4 wt.%).

**Figure 21:**
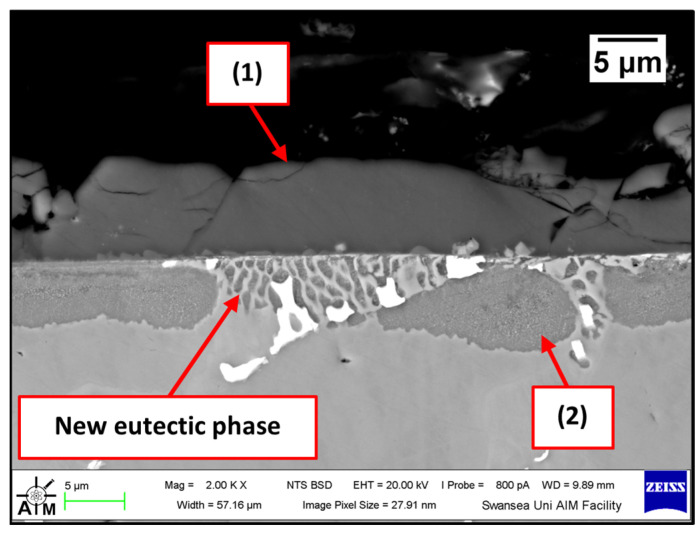
Cross-section of the Wallex4^TM^ pad specimen after exposure to Zn-Al: (1) intermetallic particles, (2) diffusion layer.

**Figure 22:**
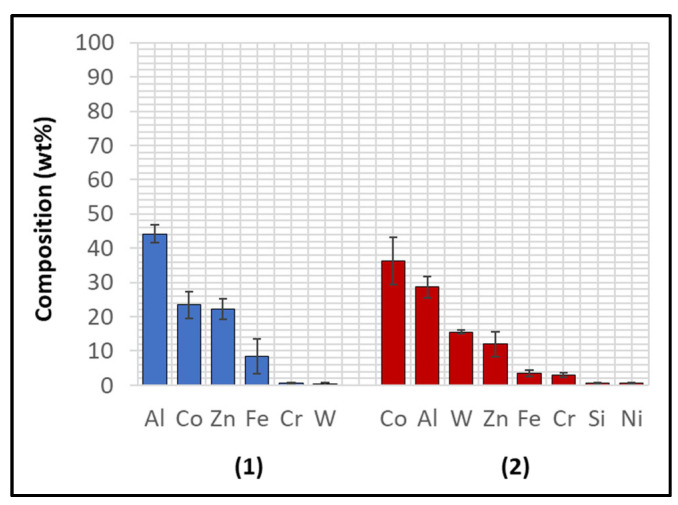
EDS analysis of the corrosion products in Wallex4^TM^ after exposure to Zn-Al. The numbers refer to the phases shown in Figure 21: (1) intermetallic compounds and (2) diffusion layer.

**Figure 23:**
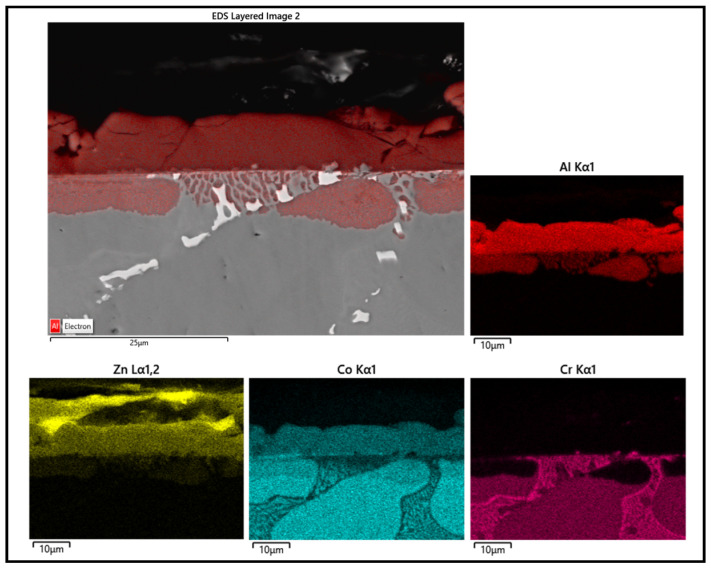
EDS mapping of the elements present in Wallex4^TM^ after testing in Zn-Al.

Several corrosion products were identified on the Wallex4^TM^ pad specimen (Figure 21), and the composition of the new phases was investigated (Figure 22). Intermetallic dross phases were found on the surface of the alloy (1), which contained Al (44.2 ± 2.6 wt.%), Co (23.4 ± 4.0 wt.%), Zn (22.3 ± 3.1 wt.%), and Fe (3.4 ± 1.0 wt.%). The results of EDS showed that the composition of the dross was similar to that of the intermetallic particles found on the bar specimen (Figure 19), although it contained 9.9 wt.% more Co and 15.2 wt.% less Fe. A subsurface reaction layer was still present (2), where both Al and Zn were detected, and the Zn content was lower compared to the dross phase. The Co and Cr content measured in this layer was found to be lower compared to the as-received specimen (Figure 7). The results of EDS analysis showed that the reaction layer contained Co (36.3 ± 7.0 wt.%) and Cr (2.9 ± 0.7 wt.%), whereas the solid solution phase of the as-received specimen contained Co (56.1 ± 0.4 wt.%) and Cr (27.8 ± 0.2 wt.%). Therefore, the analysis suggested that the depletion of these elements from the reaction layer occurred. Moreover, it was observed that the CoCrW eutectic phase present in Wallex4^TM^ (Figure 5b) reacted with the molten metal bath as highlighted in the EDS maps (Figure 23), which show evidence of Al diffusion from the bath.

**Figure 24:**
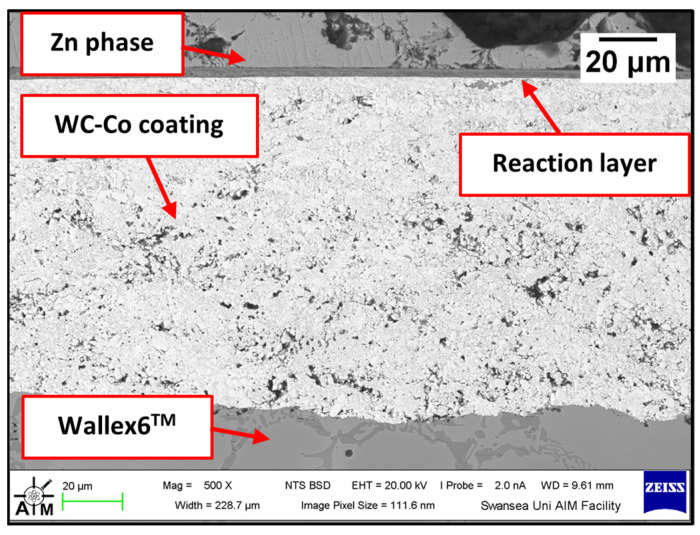
Cross-section of WC-Co/Wallex6^TM^ after dynamic testing with Wallex4^TM^ in Zn-Al-Mg.

**Figure 25:**
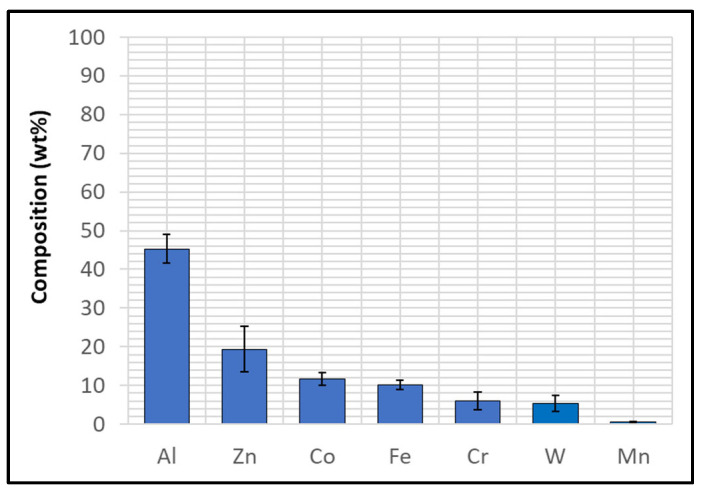
EDS analysis on the reaction layer shown in Figure 24.

A similar corrosion behaviour was observed in the samples immersed in Zn-Al-Mg. Figure 24 illustrates that reaction products developed onto the WC-Co coating (1), which were found to mainly contain Al (45.3 ± 3.7 wt.%), Zn (19.4 ± 5.8 wt.%), Co (11.7 ± 1.7 wt.%) and Fe (10.3 ± 1.2 wt.%), as shown in Figure 25. The reaction products of this specimen formed a distinct layer, and their presence confirms that the Co matrix of the thermal sprayed coating reacts with the elements present in the bath. No reaction with Mg was detected, as observed in the benchmark configuration.

**Figure 26:**
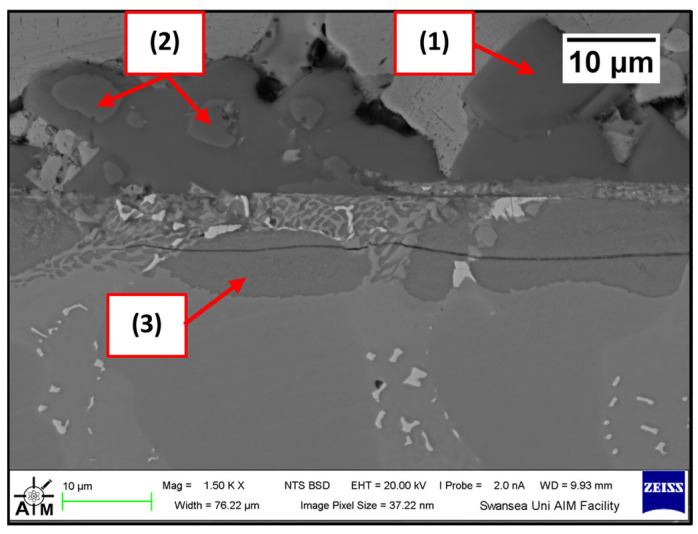
SEM image of the Wallex4^TM^ pad after dynamic testing in Zn-Al-Mg: (1) intermetallic particles, (2) possible wear debris, (3) diffusion layer.

**Figure 27:**
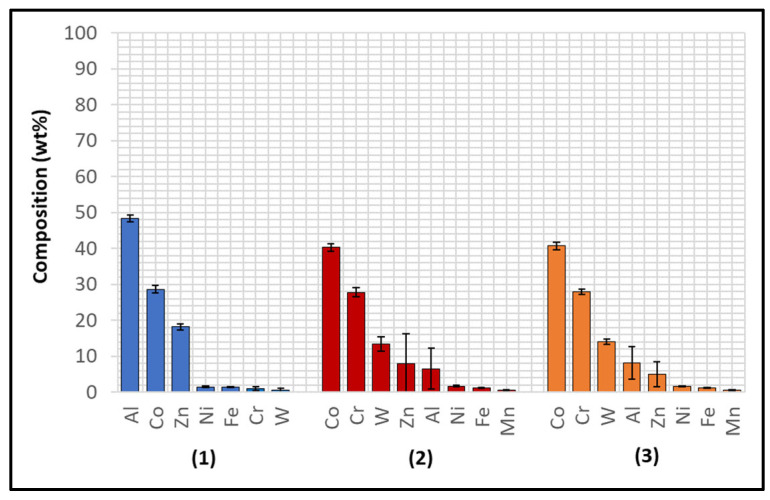
EDS analysis conducted on Wallex4^TM^ after exposure to Zn-Al-Mg. The numbers refer to the phases shown in Figure 26: (1) intermetallic particles, (2) possible wear debris, (3) diffusion layer.

Figure 26 and Figure 27 show that AlCoZn dross was again present on the Wallex4^TM^ pad specimen (1); in addition to this, CoCrW particles were present within this layer (2), which are likely wear debris from the alloy due to their composition. Al diffused into the Co solid solution phase forming a subsurface reaction layer (3), as well as in the CoCrW eutectic phases, in a similar fashion to the specimen tested in Zn-Al. The reaction layer was found to contain Al (8.22 ± 4.5 wt.%) and only small amounts of Mg (~0.1 wt.%).

**Figure 28:**
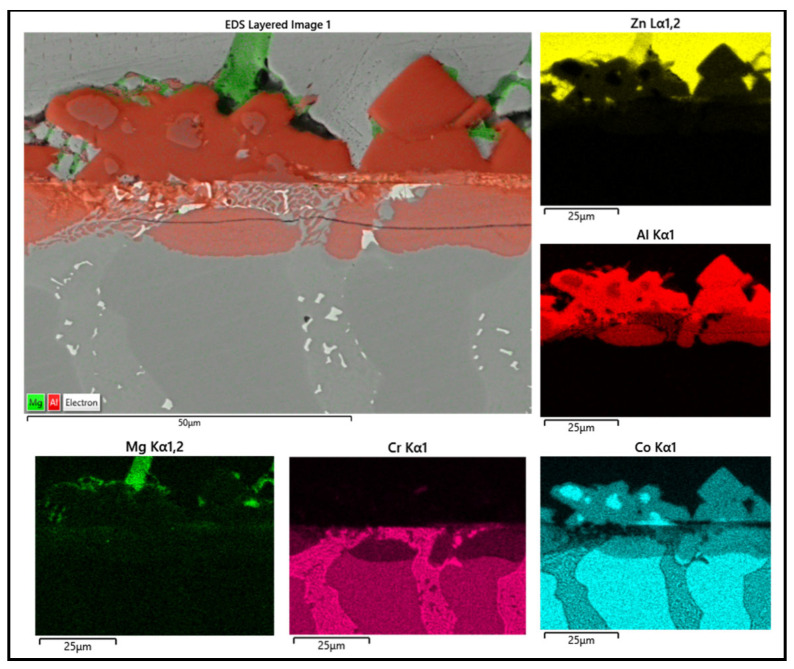
EDS mapping of the elements present in Wallex4^TM^ after testing in Zn-Al-Mg.

Similarly, EDS elemental mapping showed that mostly Al diffused into the eutectic phases from the molten metal bath (Figure 28). Overall, changing the bath from Zn-Al to Zn-Al-Mg did not alter the interactions between the materials and the liquid metal significantly. Wallex4^TM^ was corroded in both baths and WC-Co reacted with Al. Moreover, the corrosion behaviour of Wallex4^TM^ showed similarities with Wallex6^TM^, due to the similar chemical composition of the two alloys. The Co-rich solid solution phase of both alloys was attacked by molten Zn and Al forming diffusion layers and, additionally, they developed a layer of Co-Al-Fe-Zn intermetallic phases on the surface.

#### 3.2.3. SS 316L with HVOF Al_2_O_3_ and Wallex6^TM^

**Figure 29:**
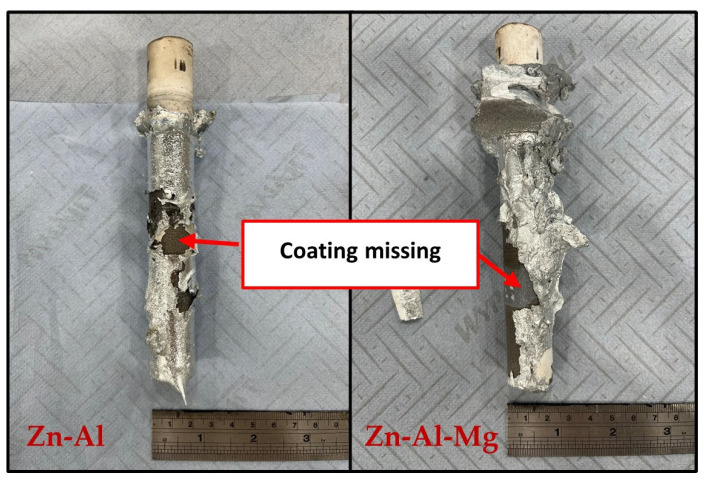
Visual inspection of the bar specimens tested in Zn-Al (**left**) and Zn-Al-Mg (**right**).

**Figure 30:**
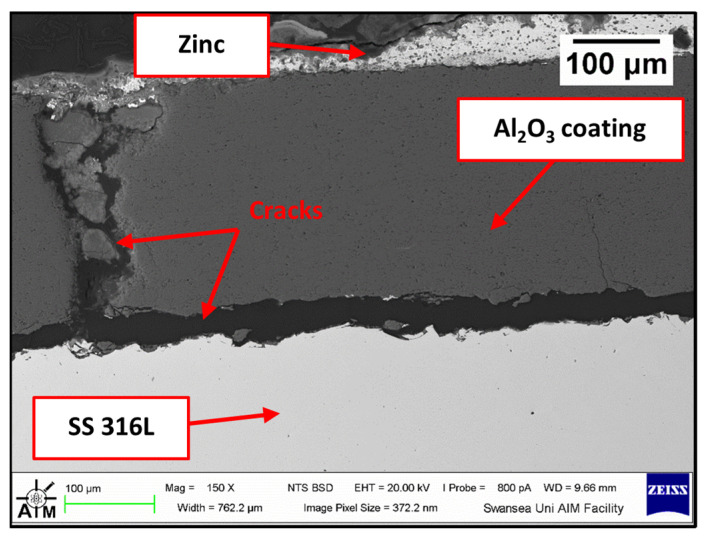
Cross-section of the Al_2_O_3_/SS 316L bar after dynamic testing in Zn-Al.

Visual inspection of the bars tested in Zn-Al and Zn-Al-Mg (Figure 29) after extraction from each molten metal bath evidenced clear signs of damage on the ceramic coatings, leaving areas of the SS 316L specimen unprotected. Examination of the bar with SEM after 48 h of testing in Zn-Al (Figure 30) confirmed that structural damage occurred in the Al_2_O_3_ coating, as both vertical and horizontal cracks are present. These cracks were not observed in the as-received specimen (Figure 3). The large horizontal crack in the SEM image shows that the coating was forced to separate from the steel bar, in a similar fashion to the mechanism observed during static testing. It was shown in a previous study that cracks and spallation of the coating can be linked to stresses developing due to the large thermal expansion mismatch between Al_2_O_3_ and SS 316L [17]. The CTEs recorded at ~465 °C for Al_2_O_3_ and SS 316L were 8.2 × 10^−6^ and 21.2 × 10^−6^ K^−1^ respectively and it is believed that this difference resulted in the formation of cracks during dynamic testing. However, despite suffering from significant damage, the ceramic coatings remained completely inert to Zn-Al. No evidence of Zn penetration through cracks and pores was observed during the examination of the cross-sections with SEM and, as a result, reaction products did not accumulate below the coating. Therefore, these observations exclude that the breakdown of the coating occurred due to the build-up of corrosion products on the base metal, suggesting that the coating spalled due to the large thermal expansion mismatch, as discussed. The surface of the coating was free from dross phases, which were observed to build up on WC-Co. This observation agrees with the findings obtained under static conditions in Zn-Al [17].

**Figure 31:**
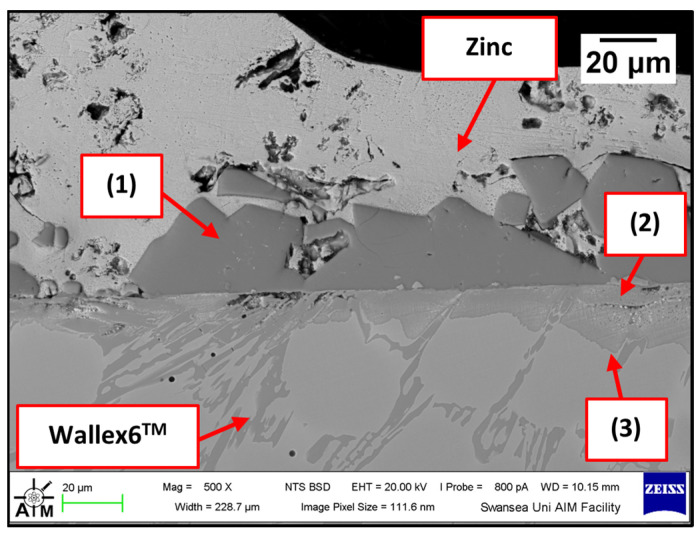
SEM image of the Wallex6^TM^ pad after dynamic testing with SS 316L/Al_2_O_3_ in Zn-Al: (1) intermetallic particles, (2,3) diffusion layer.

**Figure 32:**
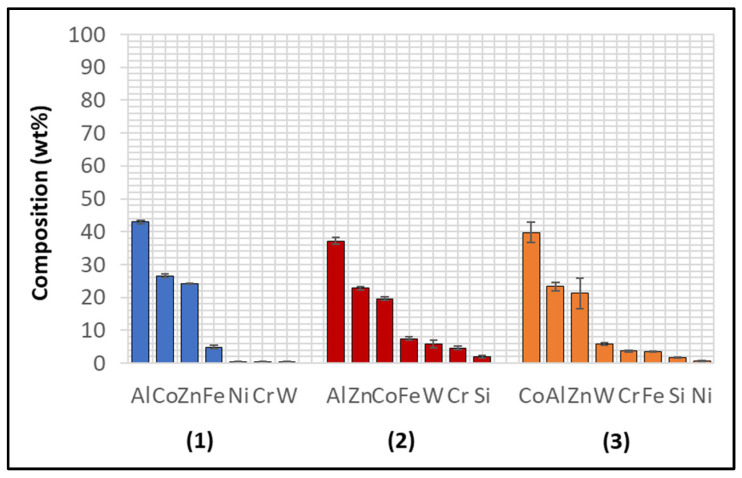
Composition of the phases present in Wallex6^TM^ after dynamic testing with SS 316L/Al_2_O_3_ in Zn-Al. The numbers refer to the phases shown in Figure 31, namely the (1) intermetallic particles and (2,3) diffusion layer.

The performance of Wallex6^TM^ was analogous to that observed in the benchmark configuration. Figure 31 shows that intermetallic particles of Al-Co-Zn-Fe composition are present on the surface of the alloy (1). Furthermore, a subsurface reaction layer developed, and the results of EDS point spectrum analysis (Figure 32) confirmed that Al was contained within this layer (37.2 ± 1.0 wt.%) and at the interface with the Co-solid solution phase of Wallex6^TM^ (23.4 ± 1.3 wt.%), which are, respectively, indicated as location (2) and (3) in Figure 31. This result confirmed that Al diffused from the melt into the alloy. In addition to this, the amount of Co at location (2) is lower (19.7 ± 0.5 wt.%) relative to the as-received sample (47.2 wt.%). On the other hand, at location (3), a Co content closer to that present in the bulk of the material was detected (39.8 ± 3.1 wt.%), suggesting that Co depletion from the diffusion layer occurred.

**Figure 33:**
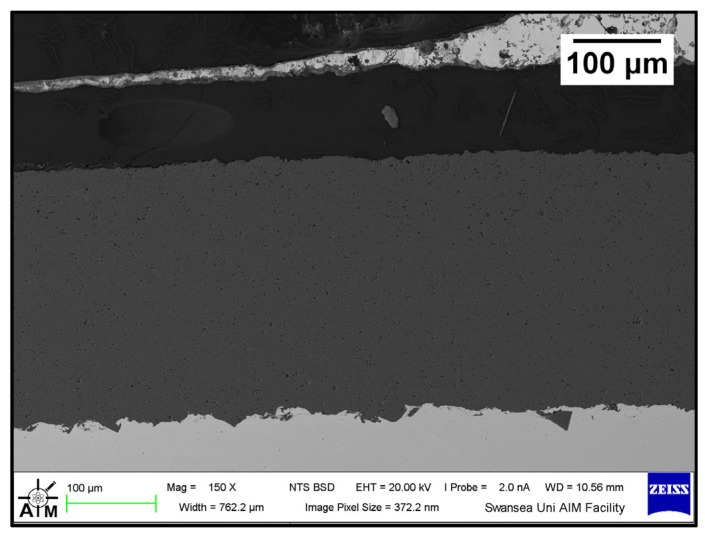
Cross-section of the Al_2_O_3_/SS 316L bar after dynamic testing in Zn-Al-Mg.

The specimens immersed in Zn-Al-Mg showed similar corrosion behaviour. The findings obtained in the previous study, in which the same Al_2_O_3_ coatings were tested under static conditions for 5 weeks, highlighted a possible reduction of Al_2_O_3_ by the Mg present in the molten metal bath [17]. However, under dynamic conditions, the SS 316L bar with Al_2_O_3_ coating remained completely unreactive after exposure to Zn-Al-Mg, as no dross build-up layer was present on the ceramic coating (Figure 33). This behaviour is believed to be linked to the shorter immersion times used in the present work.

**Figure 34:**
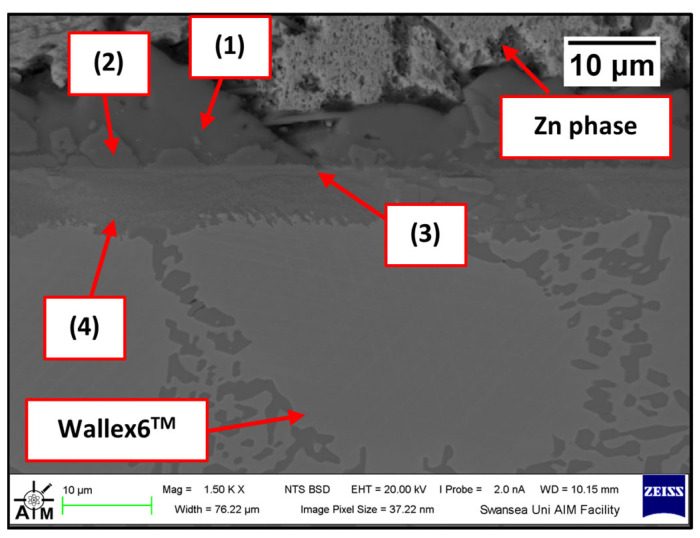
SEM image of the Wallex6^TM^ pad after dynamic testing with SS 316L/Al_2_O_3_ in Zn-Al-Mg: (1,2) intermetallic particles, (3,4) diffusion layer.

**Figure 35:**
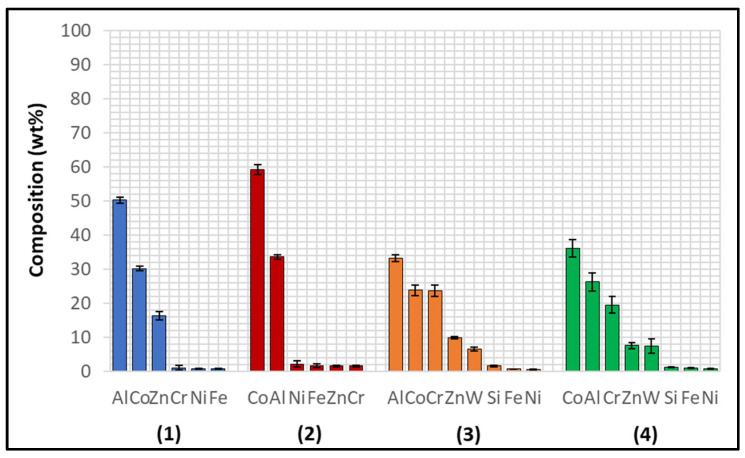
Composition of the phases in Wallex6^TM^ after dynamic testing with SS 316L/Al_2_O_3_ in Zn-Al-Mg. The numbers refer to the phases shown in Figure 34, which were detected within the (1,2) intermetallic particles and (3,4) diffusion layer.

The Wallex6^TM^ pad specimen reacted with the liquid metal bath in a similar fashion to the samples characterised previously. Within the unworn region, dross intermetallic phases are deposited onto the surface. Figure 34 shows two types of reaction products: (1) a CoAl phase containing Zn (16.3 ± 1.1 wt.%) and (2) a different CoAl phase with lower content of Zn (1.6 ± 0.3 wt.%). Co depletion from the diffusion layer occurred, as shown by the results of EDS analysis (Figure 35), which was carried out at the interface with molten metal (3) and the interface between the subsurface reaction layer and the bulk of the material (4).

#### 3.2.4. Effect of Changing Bath Composition

Overall, the results of the dynamic corrosion tests showed that the ceramic coating was the only material that remained inert during the 48 h of dynamic testing. It showed better performance compared to the WC-Co coatings, whose Co matrix reacted with the molten metal bath forming intermetallic compounds at the interface with the molten metal. Although previous studies highlighted that baths with Al content above 0.3 wt.% reduced the corrosion rate of WC-Co [26], the results of the present study showed that the coatings corroded both in Zn-Al and Zn-Al-Mg to a similar extent. The two Co-based alloys, namely Wallex6^TM^ and Wallex4^TM^, were severely corroded after exposure to molten metal. The reactivity of Wallex4^TM^ was found to not significantly differ from that of Wallex6^TM^, due to their similar chemical composition. Therefore, it did not show superior performance at the chosen testing conditions. The introduction of Mg did not affect the corrosion behaviour of the coupling pairs with the surrounding environment.

**Figure 36:**
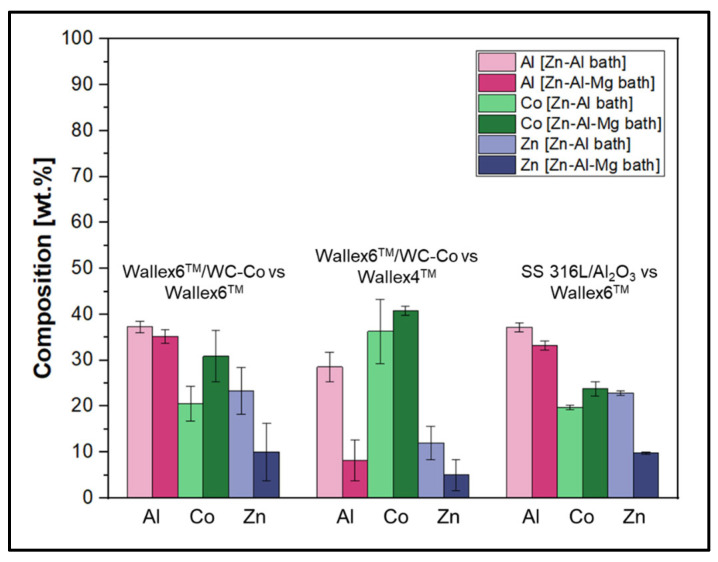
Composition of the diffusion layers developed in the pads for each material pair.

**Figure 37:**
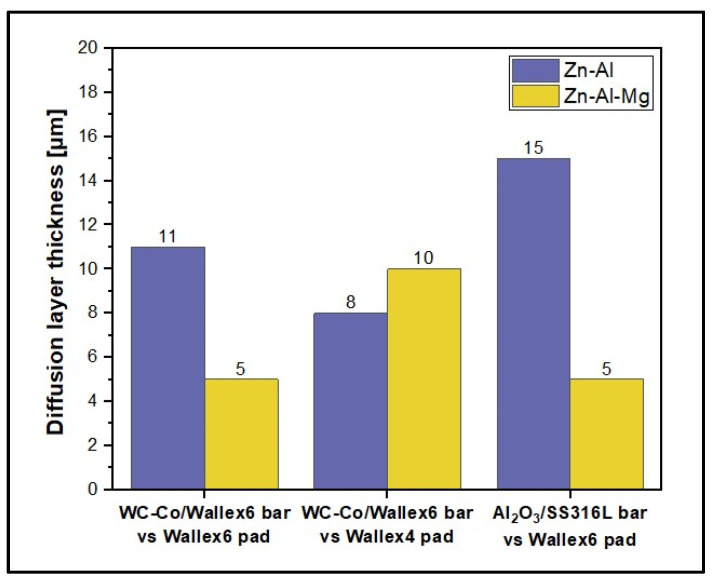
The average thickness of the diffusion layers developed in the pads for each material pair.

However, in order to investigate the effect of the higher Al content in Zn-Al-Mg, the amount of Al, Co, and Zn contained within the reaction layers of the pad specimens was tabulated for both Zn-Al (Table 3) and Zn-Al-Mg (Table 4). Figure 36 and Figure 37 show the concentration of these elements and the thickness of the diffusion layers, respectively. It was observed that the content of Al within the reaction layer decreased after changing bath composition from Zn-Al to Zn-Al-Mg. On the other hand, the reaction layers contained more Co, suggesting that less depletion of Co from these layers occurred in the Zn-Al-Mg baths. Furthermore, it was noticed that the Wallex^TM^ pads contained less Zn (~60 %) in the reaction layers after exposure to Zn-Al-Mg compared to the Zn-Al bath, suggesting that the diffusion of Zn into the alloys diminished at higher Al concentration. An analogous behaviour was observed on SS 316L specimens tested in an earlier study, due to the inhibition of the reaction between the SS 316L and the molten Zn in the bath [36]. A decrease in the depth of the diffusion layer was also observed in Wallex6^TM^, as shown in Figure 37, although no significant changes were detected in Wallex4^TM^.

### 3.3. Wear Scar Analysis

**Figure 38:**
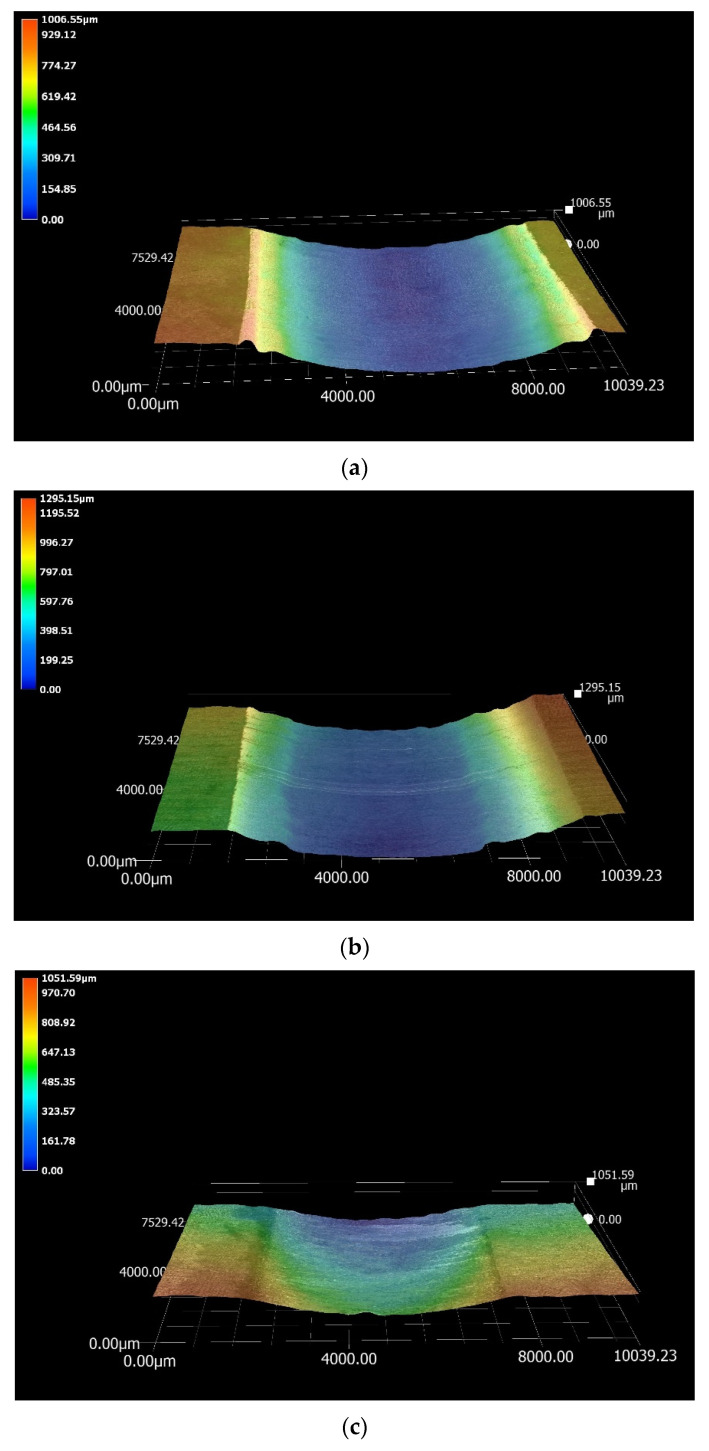
Three-dimensional (3D) imaging of the wear scars produced on the specimens tested in Zn-Al: (**a**) Wallex6^TM^ after sliding with Wallex6^TM^/WC-Co; (**b**) Wallex4^TM^ after sliding with Wallex6^TM^/WC-Co; (**c**) Wallex6^TM^ after sliding with SS 316L/Al_2_O_3_. Deep scars formed after contact with the WC-Co-coated bars, whereas a more superficial scar formed after contact with the Al_2_O_3_-coated bar specimen.

The bar displacement and the wear coefficient of each pad specimen after 48 h were calculated according to the procedures previously outlined in Section 2. Materials and Methods. The results are summarised in Table 5 for Zn-Al and Table 6 for Zn-Al-Mg. These values are plotted in Figure 38. Moreover, the percentage change of these parameters after changing the bath from Zn-Al to Zn-Al-Mg was calculated (Table 7).

The results of the wear tests conducted in Zn-Al highlighted that Al_2_O_3_/SS 316L had the lowest value of displacement (0.46 mm) and inflicted the lowest wear damage on its counterpart (*k* = 9.7 × 10^−6^ mm^3^N^−1^m^−1^). The hardness of the materials in the current bearing configuration was previously measured by Faulkner [25]. It was found that the hardness of WC was between 2100–2500 HV, which is significantly higher than the hardness of Wallex6^TM^ (400 HV) and Wallex4^TM^ (570 HV). As a result, it is believed that the WC-Co coatings inflicted severe wear damage to their softer counterparts on the contact surface, due to the greater hardness of the WC particles. This observation aligns with the findings of Zhang [37], who reported that WC particles could easily scar the surface of Stellite alloys. The hardness of Al_2_O_3_ was measured to be approximately 1680 HV [17] and, therefore, it inflicted less wear damage on its counterpart.

However, previous studies demonstrated that the wear process of materials in liquid Zn is complex, due to the chemical reactions with the molten metal bath and the complexity of the intermetallic compounds. For this reason, surface damage or removal of materials during sliding contact can involve multiple mechanisms. It is reported that wear debris can form following the breakup of the materials as well as of the intermetallic compounds. Wear debris was found to react with the elements present in the molten metal, forming particles that could easily plough the contacting surfaces [37,38]. In this work, the CoAl phases observed on the surface of WC-Co were absent on the Al_2_O_3_ coating, due to the inertness of the ceramic material. The CoAl phase is hard and can groove the bearing surfaces [30]. For this reason, it is theorised that the absence of Co-based aluminides on the Al_2_O_3_ coating contributed to reducing the wear damage on the Wallex6^TM^ counterpart. Figure 38 illustrates a comparison of the wear scars after testing in Zn-Al with 3D imaging.

**Figure 39:**
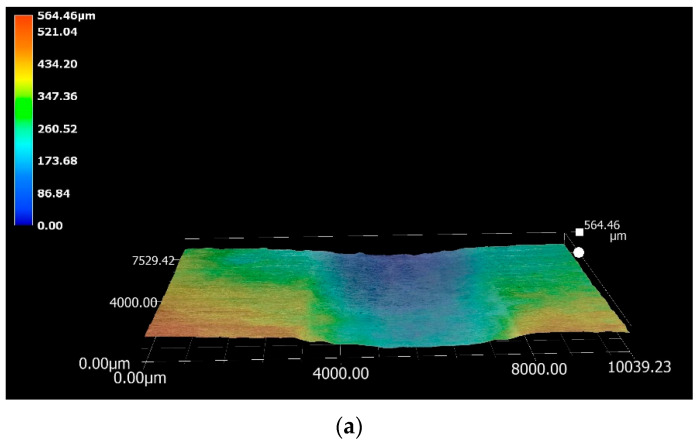

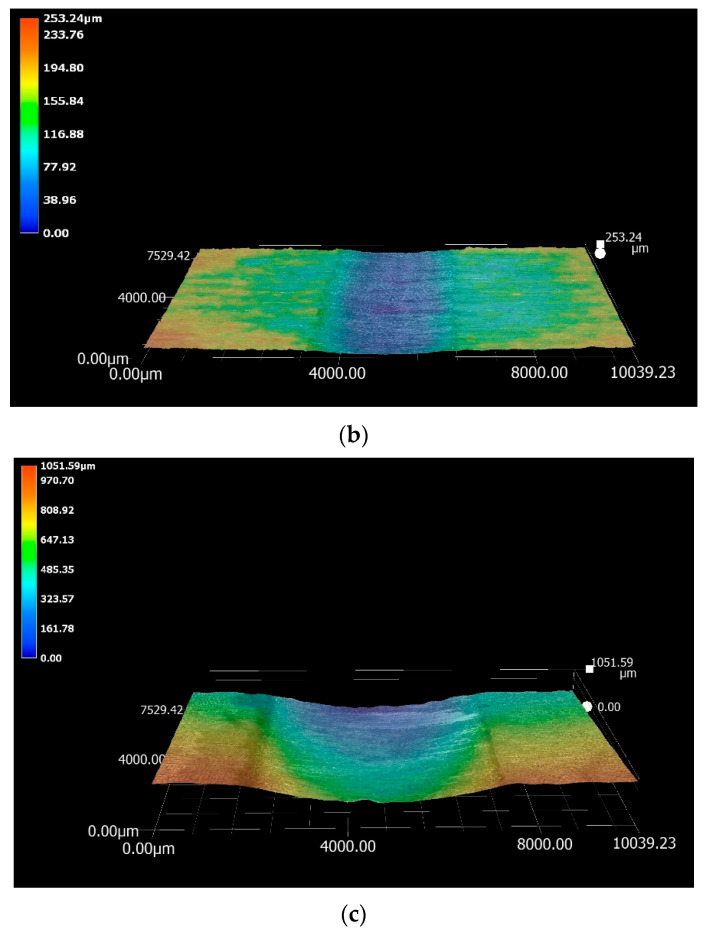
Three-dimensional (3D) imaging of the wear scars produced on the pad specimens tested in Zn-Al-Mg: (**a**) Wallex6^TM^ after sliding with Wallex6^TM^/WC-Co; (**b**) Wallex4^TM^ after sliding with Wallex6^TM^/WC-Co; (**c**) Wallex6^TM^ after sliding with SS 316L/Al_2_O_3_. The scars are more superficial compared to those formed in the Zn-Al bath.

The measurements of displacement and wear coefficients obtained for the specimens tested in Zn-Al-Mg revealed that the Wallex4^TM^ pad exhibited the highest wear resistance (0.1 mm displacement, *k* = 1.3 × 10^−6^ mm^3^N^−1^m^−1^), although less wear damage was observed in all the pad specimens immersed in Zn-Al-Mg compared to Zn-Al, as shown in Table 7. Similar reaction products formed in the two molten metal baths; however, the composition of the reaction layers formed at high Al concentration was found to vary from that measured on the samples tested in Zn-Al. It was earlier explained that the Wallex6^TM^ and Wallex4^TM^ pads tested in Zn-Al-Mg exhibited less corrosion by molten Zn after 48 h of exposure to the liquid metal. It is reported that during the rotation of submerged pot roll bearings, cracks initiate in the reaction layer and at its interface with the bulk of the material. This process is followed by the detachment of the corrosion products and the bearing materials, resulting in the formation of wear debris, which can inflict further damage to the contact surfaces [37]. Therefore, it is plausible that the specimens submerged in Zn-Al-Mg displayed less wear damage due to their limited corrosion relative to the Zn-Al bath, which reduced the likelihood of breakage of the corrosion products and, consequently, the rate of material removal at the testing conditions chosen for the experiments. The higher wear resistance exhibited in Zn-Al-Mg can be visualised with the 3D images of the contacting surfaces (Figure 39).

## 4. Conclusions

Dynamic wear testing in molten Zn-Al and Zn-Al-Mg was conducted to assess the corrosion behaviour and wear resistance of three material pairs using a bespoke wear testing rig. The following were conducted:

- WC-Co coatings protected the underlying Wallex6^TM^ base metal from the attack of liquid Zn alloy, although they developed intermetallic compounds which mainly contained Al-Co-Zn-Fe.
- Al_2_O_3_ coatings remained completely inert in liquid Zn alloy. Cracking and spallation occurred due to the large thermal expansion mismatch with the underlying SS 316L base metal.
- Wallex6^TM^ and Wallex4^TM^ were severely corroded in molten metal, showing evidence of Zn and Al diffusion within 5–15 µm from the surface.
- Changing the bath chemistry from Zn-Al to Zn-Al-Mg reduced the diffusion of Zn into Wallex6^TM^ and Wallex4^TM^ by approximately 60%, leading to less wear damage.
- Out of the three material couplings investigated in this study, minimal wear damage in both Zn-Al (*k* = 9.7 × 10^−6^ mm^3^N^−1^m^−1^) and Zn-Al-Mg (*k* = 3.6 × 10^−6^ mm^3^N^−1^m^−1^) was only obtained by pairing Wallex6^TM^ with Al_2_O_3_/SS 316L.

The results of dynamic testing confirmed that the introduction of ceramic materials has the potential to improve the performance of galvanising pot roll bearings. However, future research should focus on reducing the thermal expansion mismatch to prevent cracking in the ceramic coatings.

## Figures and Tables

**Table 1:** Material couplings chosen for dynamic testing.

Material Pair No.	Bar Material	Pad Material
1	Wallex6^TM^ coated with HVOF WC-Co	Wallex6^TM^
2	Wallex6^TM^ coated with HVOF WC-Co	Wallex4^TM^
3	SS 316L coated with HVOF Al_2_O_3_	Wallex6^TM^

**Table 2:** Composition of Wallex6^TM^ and Wallex4^TM^.

Alloy	%Fe	%Cr	%Ni	%Mn	%Si	%Co	%C	%W	%Mo
Wallex6	2.3	28.4	2.6	0.8	1	BAL	1.2	3.8	0.8
Wallex4	1.7	30.4	2.6	0.6	0.8	BAL	0.9	14.3	1.5

**Table 3:** The main composition of the reaction layers of the pads after testing in Zn-Al.

Coupling Pair	Pad Material	Al [wt.%]	Co [wt.%]	Zn [wt.%]
Wallex6^TM^/WC-Co vs. Wallex6^TM^	Wallex6^TM^	37.3 ± 1.2	20.6 ± 3.8	23.3 ± 5.1
Wallex6^TM^/WC-Co vs. Wallex4^TM^	Wallex4^TM^	28.6 ± 3.2	36.3 ± 7.0	12.0 ± 3.6
SS 316L/Al_2_O_3_ vs. Wallex6^TM^	Wallex6^TM^	37.2 ± 1.0	19.7 ± 0.5	22.8 ± 0.5

**Table 4:** The main composition of the reaction layers of the pads after testing in Zn-Al-Mg.

Coupling Pair	Pad Material	Al [wt.%]	Co [wt.%]	Zn [wt.%]
Wallex6^TM^/WC-Co vs. Wallex6^TM^	Wallex6^TM^	35.2 ± 1.5	30.9 ± 5.3	10.0 ± 6.2
Wallex6^TM^/WC-Co vs. Wallex4^TM^	Wallex4^TM^	8.2 ± 4.5	40.8 ± 1.0	5.0 ± 3.4
SS 316L/Al_2_O_3_ vs. Wallex6^TM^	Wallex6^TM^	33.2 ± 1.0	23.8 ± 1.6	9.8 ± 0.3

**Table 5:** Wear coefficient and displacement after testing in Zn-Al.

Coupling Pair	Pad Material	Wear Coefficient k × 10^−6^ [mm^3^N^−1^m^−1^]	Displacement [mm]
Wallex6^TM^/WC-Co vs. Wallex6^TM^	Wallex6^TM^	26.3	0.80
Wallex6^TM^/WC-Co vs. Wallex4^TM^	Wallex4^TM^	30.9	1.01
SS 316L/Al_2_O_3_ vs. Wallex6^TM^	Wallex6^TM^	9.7	0.46

**Table 6:** Wear coefficient and displacement after testing in Zn-Al-Mg.

Coupling Pair	Pad Material	Wear Coefficient k × 10^−6^ [mm^3^N^−1^m^−1^]	Displacement [mm]
Wallex6^TM^/WC-Co vs. Wallex6^TM^	Wallex6^TM^	4.5	0.21
Wallex6^TM^/WC-Co vs. Wallex4^TM^	Wallex4^TM^	1.3	0.10
SS 316L/Al_2_O_3_ vs. Wallex6^TM^	Wallex6^TM^	3.6	0.21

**Table 7:** Effect of changing bath composition on the wear coefficient and displacement.

Coupling Pair	Pad Material	Percentage Decrease Wear Coefficient	Percentage Decrease Displacement
Wallex6^TM^/WC-Co vs. Wallex6^TM^	Wallex6^TM^	83%	74%
Wallex6^TM^/WC-Co vs. Wallex4^TM^	Wallex4^TM^	96%	90%
SS 316L/Al_2_O_3_ vs. Wallex6^TM^	Wallex6^TM^	63%	54%

## Data Availability

Data are contained within the article.

## References

[1] Gossuin T., Moreas G. Cleanliness Measurement by Innovative Libs Method. Proceedings of the 12th International Conference on Zinc and Zinc Alloy Coated Steel Sheet.

[2] Beentjes P., Bottema J., Salgin B., Vrenken J. An Innovative GI with Improved Galling and Surface Properties for Exposed Automotive Applications. Proceedings of the 12th International Conference on Zinc and Zinc Alloy Coated Steel Sheet.

[3] Chakraborty A., Ghassemi-Armaki H. Evaluation of Initiation and Propagation of LME Cracks on the Galvanised 3G-AHSS Using Interrupted Resistance Spot-Welding Method. Proceedings of the 12th International Conference on Zinc and Zinc Alloy Coated Steel Sheet.

[4] Sohn I.-R., Kim T.-C., Ju G.-I., Kim M.-S., Kim J.-S. (2021). Anti-Corrosion Performance and Applications of PosMAC^®^ Steel. Corros. Sci. Technol..

[5] Kim S., So S., Park J., Kim T., Han S., Park S., Kim H.-y., Kim M., Paik D. (2024). Characteristics of Hot-Dip Znmgal Coatings with Ultra-High Corrosion Resistance. Corros. Sci. Technol..

[6] Tang N.Y., Daniel L., Zhang K. (2010). Performance of Submerged Hardware in Continuous Galvanizing. Corros. Sci. Technol..

[7] Zhang K., Tang N.Y., Goodwin F. Research and development of pot bearings in continuous galvanizing. Proceedings of the 6th International Conference on Zinc and Zinc Alloy Coated Steel Sheet.

[8] Zhang K. (2005). Effects of test conditions on the tribological behaviour of a journal bearing in molten zinc. Wear.

[9] Marder A., Goodwin F. (2023). The Metallurgy of Zinc Coated Steels.

[10] Escott L.J., Penney D.J., Das A., Thomas D. Effects of Heat Treatments on the Morphology and Mechanical Properties of a CoCrW Alloy for Hot Dip Galvanising Applications. Proceedings of the 12th International Conference on Zinc and Zinc Alloy Coated Steel Sheet.

[11] Faulkner R., Penney D., Bright M. Offline Simulation of Galvanising Bath Journal Bearings as a Cost Effective Solution to Improve Line Performance and Mitigate Risk. Proceedings of the 12th International Conference on Zinc & Zinc Alloy Coated Steel Sheet.

[12] Nag A., Bhadu M.K., Bijalwan P.K., Pathak A.S. (2021). Investigation of selected HVOF and plasma sprayed coatings for sustained performance in molten zinc. Corros. Sci..

[13] Dong Y., Yan D., He J., Zhang J., Li X. (2006). Degradation behaviour of ZrO_2_–Ni/Al gradient coatings in molten Zn. Surf. Coat. Technol..

[14] Jarosinski W., Quets J., Wang D., Belov V., Klryman A.S. (2013). Thermal Spray Coated Rolls for Molten Metal Baths.

[15] Liu X., Zhao X., Yang F. (2020). Room-Temperature and High-Temperature Wear Behaviors of As-Sprayed and Annealed Cr_3_C_2_-25NiCr Coatings Prepared by High Velocity Air-Fuel Spraying. Coatings.

[16] Wang Q., Zhang J.W., Zhang L., Liu H.J., Zeng C.L. (2019). Stability investigation of electrodeposited zirconium diboride ceramic coatings in molten zinc. Mater. Corros..

[17] Alparone G.P., Penney D., Sullivan J., Edy J., Mills C. (2024). Al_2_O_3_ Coatings for Protection of Stainless Steel 316L against Corrosion in Zn-Al and Zn-Al-Mg. Coatings.

[18] Matthews S.J., James B. (2010). Review of Thermal Spray Coating Applications in the Steel Industry: Part 2—Zinc Pot Hardware in the Continuous Galvanizing Line. J. Therm. Spray Technol..

[19] Shi Y., Tong J., Zheng Q., Rao S., Jin H., Rao S. (2018). Analysis About the Failure Mechanism of the Sleeves in Sink Roll System. J. Fail. Anal. Prev..

[20] Liu X., Barbero E., Xu J., Burris M., Chang K.-M., Sikka V. (2005). Liquid metal corrosion of 316L, Fe_3_Al, and FeCrSi in molten Zn-Al baths. Metall. Mater. Trans. A.

[21] Jiang W., Wang W. (2019). Sliding Bearing Material Testing Machine under High Temperature Corrosion Conditions. IOP Conf. Ser. Mater. Sci. Eng..

[22] Zhang K., Battiston L. (2002). Friction and wear characterization of some cobalt- and iron-based superalloys in zinc alloy baths. Wear.

[23] Yin L., Zhao K., Ding Y., Wang Y., He Z., Huang S. (2022). Effect of hBN addition on the fabrication, mechanical and tribological properties of Sialon materials. Ceram. Int..

[24] Pulsford J., Venturi F., Pala Z., Kamnis S., Hussain T. (2019). Application of HVOF WC-Co-Cr coatings on the internal surface of small cylinders: Effect of internal diameter on the wear resistance. Wear.

[25] Faulkner R. (2020). Material Selection and Mechanical Design of Submerged Galvanising Bath Journal Bearings. Ph.D. Thesis.

[26] Seong B.G., Hwang S.Y., Kim M.C., Kim K.Y. (2001). Reaction of WC–Co coating with molten zinc in a zinc pot of a continuous galvanizing line. Surf. Coat. Technol..

[27] Tani K., Tomita T., Kobayashi Y., Takatani Y., Harada Y. (1994). Durability of Sprayed WC/Co Coatings in Al-added Zinc Bath. ISIJ Int..

[28] Zhang K., Tang N.-Y. (2004). Reactions of Co based and Fe based superalloys with a molten Zn–Al alloy. Mater. Sci. Technol..

[29] Zhang K. (2003). Wear of cobalt-based alloys sliding in molten zinc. Wear.

[30] Zhang K., Tang N.Y. (2003). On the wear of a cobalt-based superalloy in zinc baths. Metall. Mater. Trans. A.

[31] Tang N.-Y. (2000). Determination of liquid-phase boundaries in Zn-Fe-Mx systems. J. Phase Equilibria.

[32] Ilinca F., Ajersch F., Baril C., Goodwin F.E. (2007). Numerical simulation of the galvanizing process during GA to GI transition. Int. J. Numer. Methods Fluids.

[33] McDermid J.R., Kaye M.H., Thompson W.T. (2007). Fe Solubility in the Zn-Rich Corner of the Zn-Al-Fe System for Use in Continuous Galvanizing and Galvannealing. Metall. Mater. Trans. B.

[34] Kuperus M. (2018). The Delamination Process of the Dross Build-Up Structure on Submerged Hardware in Zn-Al and Zn-Mg-Al Baths: 564 An Empirical Study. Master’s Thesis.

[35] McDermid J., Baril É., Goodwin F. Galvanizing Bath Management During Galvanize to Galvanneal and Galvanneal to Galvanize Product Transitions. Proceedings of the 6th International Conference on Zinc and Zinc Alloy Coated Steel Sheet.

[36] Alparone G.P., Penney D., Mills C. Static Immersion of Ceramics in Zn-Al and Zn-Al-Mg for Optimised Galvanising Bath Bearings. Proceedings of the 13th International Conference on Zinc and Zinc Alloy Coated Steel Sheet.

[37] Zhang K. (2005). On the Selection of Materials for Improved Performance of Pot Bearings. AISTech—Iron Steel Technol. Conf. Proc..

[38] Zhang K., Battiston L., Goodwin F. Friction and Wear Characteristics of Materials in Molten Zinc. Proceedings of the 5th International Conference on Zinc and Zinc Alloy Coated Steel Sheet.

